# Fermi-surface-free superconductivity in underdoped (Bi,Pb)(Sr,La)_2_CuO_6+*δ*_ (Bi2201)

**DOI:** 10.1038/srep09739

**Published:** 2015-06-18

**Authors:** Peter Mistark, Hasnain Hafiz, Robert S. Markiewicz, Arun Bansil

**Affiliations:** 1 Department of Physics, Northeastern University, Boston

## Abstract

*Fermi-surface-free superconductivity* arises when the superconducting order pulls down spectral weight from a band that is completely above the Fermi energy in the normal state. We show that this can arise in hole-doped cuprates when a competing order causes a reconstruction of the Fermi surface. The change in Fermi surface topology is accompanied by a characteristic rise in the spectral weight. Our results support the presence of a trisected superconducting dome, and suggest that superconductivity is responsible for stabilizing the (*π*,*π*) magnetic order at higher doping.

Recent ARPES studies on the pnictides adduce that the superconducting (SC) transition temperature *T*_*C*_ depends sensitively on details of the band structure and Fermi surface (FS)^1^. In particular, proximity of the FS to a band edge and the associated Van Hove singularity (VHS) correlates with significantly enhanced *T*_*C*_'s. An investigation of Ba_1−*x*_K_*x*_Fe_2_As_2_^2^ indicates that when a band edge approaches the Fermi energy, superconductivity can be observed even before the band crosses the Fermi energy. Bang^2^ suggests that this *Fermi-surface-free superconductivity* (FS-free SC) is driven by the shadow bands resulting from the symmetrization of the spectral weight around the Fermi energy and the formation of a related shadow gap in the Bardeen-Cooper-Schrieffer (BCS) theory of superconductivity.

With this information, the question we ask is: Does this effect also arise in the single-band case of the hole-doped cuprates when the band is split by magnetic order? To answer this question we take our inspiration from the change in FS topology of the electron-doped cuprates. In the electron doped system it is well known that the (*π*,*π*) antiferromagnetic (AF) order can induce two distinct *topological transitions* (TT's) with doping^3^,^4^. At half-filling, the AF order splits the band into upper and lower magnetic bands (U/LMBs), and low electron doping moves the Fermi energy into the bottom of the UMB. As doping increases, the LMB moves up in energy and eventually crosses the Fermi energy leading to the emergence of hole pockets around (*π*/2,*π*/2). This is the first topological transition (TT1) in this system. The second topological transition (TT2) occurs at higher doping when the electron and hole pockets merge into the single large FS of the paramagnetic state.

In this paper we show that a transition similar to TT1 can occur in hole doped cuprates such as (Bi,Pb)_2_(Sr,La)_2_CuO_6+*δ*_ (Bi2201)^5^. This transition is however different in that the first holes now enter the LMB, so that the transition occurs when the UMB moves down in energy and crosses the Fermi energy, introducing electron pockets around (*π*,0)^6^,^7^. Thermopower studies^8^,^9^ have suggested the existence of electron pockets appearing in hole-doped cuprates in the underdoped regime. Also, the remarkable finding of the ARPES experiment of Kondo *et al.* [5] is that there is spectral weight in the (*π*,0) region, starting at low doping, suggestive of the appearance of an electron pocket at (*π*,0). To preserve the analogy with TT1 in the electron doped cuprates^3^,^4^, we use a (*π*,*π*)-AF model to investigate the emergence of electron pockets in Bi2201 and Bi2212^10^, although a resonant-valence-bond spin-liquid model^11^ (YRZ) would yield similar results^8^. Near this transition we find evidence for FS-free SC, consistent with many recent experiments^8^,^9^,^12^,^13^,^14^ suggesting that FS-free SC in Bi2201 may be a general property of the cuprates.

## Results

Our analysis is based on a one band mean-field Hubbard model with competing AF and SC orders, which we have invoked previously in connection with electron-doped cuprates^15^. Using quasi-particle GW (QP-GW)^16^,^17^ self-energy corrections, we have shown that this model provides a reasonable description of many salient features of the electronic spectra of the cuprates as observed in ARPES^18^ and other spectroscopies^19^,^20^. The Hamiltonian is


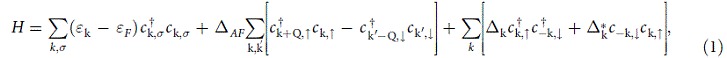


where 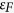 is the Fermi energy, 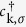 and 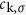 are the creation and annihilation operators for an electron of momentum k and spin 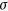. 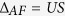 is the AF gap parameter with *U* denoting the Hubbard U and *S* the staggered magnetization. 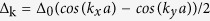 is the d-wave SC gap, where Δ_0_ is the maximum gap, and 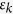 gives the bare band dispersion as


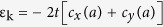



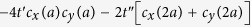



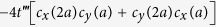



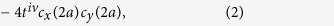


where *t*^*i*^ are the hopping parameters, 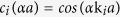, and *a* is the lattice constant. The hopping parameters used here are based on photoemission experiment^21^, for which the VHS in the AF + SC system is found around *x *= 0.37. The AF gap in Eq. (1) splits the quasiparticle spectrum into the UMB (*v *= +) and the LMB (*v *= −), which are further split by superconductivity, yielding dispersions


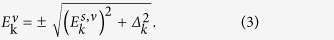


Here, 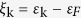 and 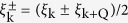. 
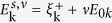
 describes the quasiparticles in the non-superconducting state with AF order, and 
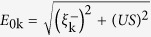
. The diagonalization results in coherence factors for the two bands as follows:


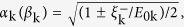



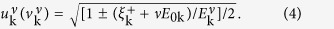


We can write equations for the gap Δ_0_ and the staggered magnetization, *S*, in terms of the coherence factors as


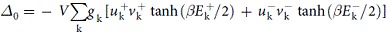



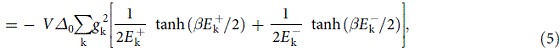



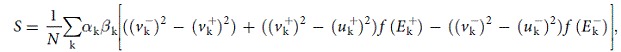



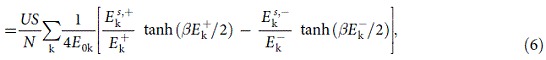


where 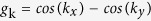.

**Figure 1:**
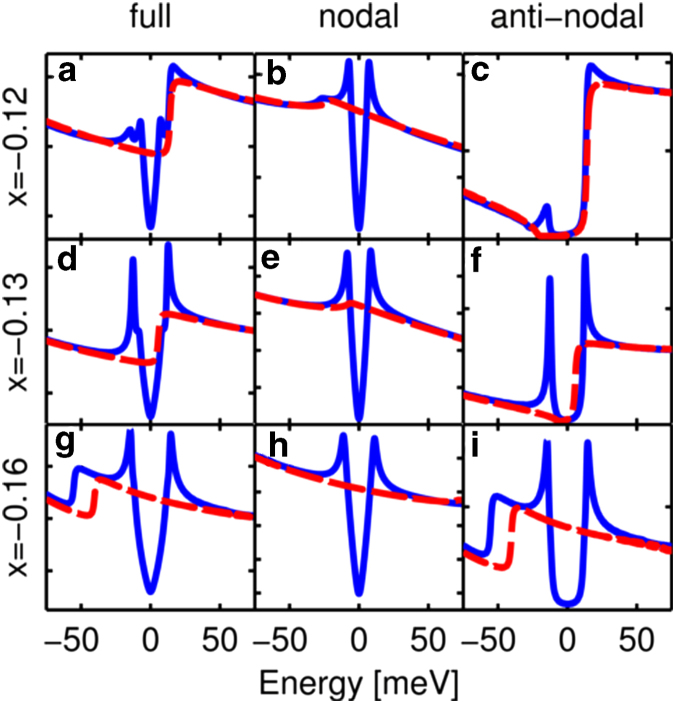
TT1 and FS-free superconductivity in the DOS. (**a**-**i**): DOS for the AF + SC system (blue curves) and the AF only system (red dashed curves). The dopings shown are *x* = 0.12 (**a**-**c**) *x* = 0.13 (**d**-**f**), and *x* = 0.16 (**g**-**i**) The first column (a,d,g) shows the full DOS. The second column (b,e,h) is the DOS calculated only in the nodal region. Similarly, the third column (c,f,i) is the DOS in the antinodal region. 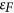 is defined to be the energy zero.

We begin our analysis by illustrating how FS-free SC arises within our model by considering the hole doped Bi2201 system using the tight-binding parameters: *t* = 0.22 eV, 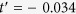 eV, 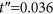 eV, 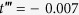 eV, and 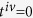 eV, as determined from fits to experimental data in Ref. 21. The leftmost column of Fig. 1 shows how the density of states (DOS) varies in the vicinity of TT1 as a function of hole-doping *x*. In the absence of SC order (red dashed curves), the bottom of the UMB shows up as a step increase in the DOS at an energy that decreases with doping, signaling the decrease of the AF gap, Δ_AF_. TT1 occurs at *x* = 0.138 in Bi2201 when the step edge crosses 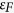. The transition would occur between the dopings shown in frames (d) and (g) of Fig. 1. In the central and right hand rows of Fig. 1, the DOS is separated into nodal and antinodal contributions, showing that the bottom of the UMB lies close to the antinodal direction (*π*,0). [We define the nodal region as the part of the first Brillouin zone (BZ) contained in 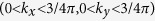, while the antinodal region is the remainder of the first BZ.]

When SC order is turned on, coherent peaks and a d-wave gap appear in the DOS (solid blue lines in Fig. 1). While the gap in the nodal region depends only weakly on doping, as seen in the central column in Fig. 1, evolution of the antinodal SC state with doping is more complex. At the highest doping, *x* = 0.16, there is a well-formed antinodal electron pocket with a conventional SC gap (bottom row of Fig. 1). Note however that when superconductivity turns on, the bottom of the UMB is shifted to lower energy. The SC gap is nearly symmetric, except for excess weight below 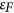. At *x* = 0.13 (middle row), the situation is completely changed. The bottom of the UMB is no longer seen clearly, but the SC gap now has two components, an inner gap and an outer gap. Note that the gap asymmetry has now reversed, with more weight lying above 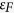. The interpretation of these features can be clarified with reference to the *x* = 0.12 results in the top row of Fig. 1. Here the anisotropy is larger, indicating that the outer peak above *x* = 0.13 is derived from the bottom of the UMB. Decomposing the DOS, we see that the inner gap arises from the nodal FS, Fig. 1(b,e), and the outer gap from the antinodal region, Fig. 1(c,f), even though the UMB would be entirely above the Fermi level in the absence of SC order. This is the essence of the phenomenon of *FS-free SC.* In this case, FS-free SC exists at the antinodes simultaneously with a standard SC gap at the node resulting in the outer and inner gap seen in the DOS as discussed. Notably, here FS-free SC acts as a precursor to TT1.

**Figure 2:**
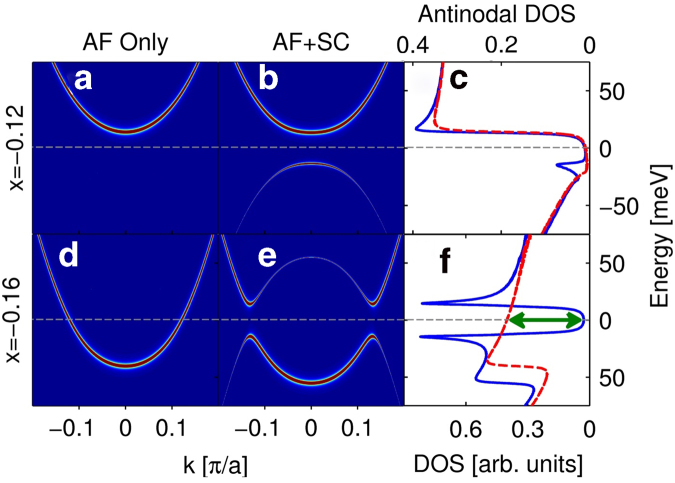
TT1 and FS-free superconductivity in dispersion. (**a**) Cut in momentum space from *k* = (−0.2*π*,*π*) to (0.2*π*,*π*) for the spectral weight in the AF ordered system at *x* = 0.12. At this doping, (**b**) shows the same cut in the presence of AF + SC order. (**c**) Antinodal DOS of the system in (**a**), red dashed curve, and (**b**), blue curve. The second row (**d**-**f**) is the same as the first row (**a**-**b**) except that this row refers to *x* = 0.16. Gray dashed lines mark 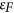. Width of the green double arrow is proportional to the AN pair spectral weight, which is the gapped spectral weight for the SC ordered system.

Further insight is obtained from Fig. 2, which presents spectral weight maps for cuts along *k* = (−0.2*π*,*π*) to (0.2*π*,*π*) and compares them to the antinodal DOS. Frames (a) and (d) of Fig. 2 show the location of the UMB with only AF order present for *x* = 0.12 and *x* = 0.16. The UMB crosses 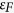 (gray dashed lines) as doping is increased. The bottom of the UMB is seen in the antinodal DOS, reproduced from Fig. 1 in Fig. 2(c,f), as a step edge in the red-dashed curves. When SC order is turned on, spectral weights in Fig. 2(a,d) develop a gap around 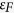, shown in Fig. 2(b,e), respectively, shown in the antinodal DOS as the blue curves in Fig. 2(c,f). To quantify the development of the AF and SC orders, we define the *gapped spectral weight* as the magnitude of the decrease in the antinodal DOS as temperature is lowered when the corresponding order turns on^5^. Thus, Fig. 2(f) shows the change in the antinodal DOS at 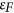 between the AF + SC and AF only ordered states (indicated by a double green arrow). Fig. 2(c) shows that the gapped spectral weight is zero before TT1 takes place, where the system is in the FS-free SC regime.

**Figure 3:**
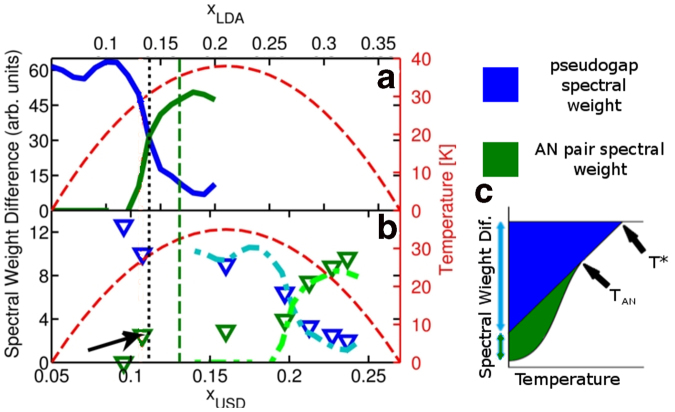
Theoretical and experimental pseudogap spectral weight and AN pair spectral weight. Theoretical (**a**) and experimental^5^ (**b**) pseudogap spectral weight [blue curve in (**a**) and triangles in (**b**)], and AN pair weight in Bi2201 [green curve in (**a**) and triangles in (**b**)]. The red dashed curve shows the SC dome, *T*_*C*_(*x*), with temperature on the right hand vertical axis. The values for *T*_*C*_ are estimated as Δ_*SC*_ = 5*k*_*B*_*T*_*C*_^36^ and the SC dome is assumed to be parabolic, given by Δ_0_ = 0.01637[1 − 39.0625(0.21 − *x*_*LDA*_)^2^]. Vertical lines spanning (**a**) and (**b**) represent the beginning of TT1 as determined in this work (black dotted line), thermopower^8^ (green dashed line), and STM^28^ (orange dot-dashed line) experiments. The black arrow in (**b**) points to the onset of AN weight in experimental data. Light blue and green dot-dashed lines in (**b**) represent our data in (**a**) shifted by *x*_*USD*_ = 0.0903 and scaled by 5/30. The difference between *x*_*LDA*_ and *x*_*USD*_ is addressed in the methods section. (**c**) Schematic representation of the experimental data form Kondo *et al.*^5^, showing spectral weight differences as a function of temperature at an arbitrary doping. This illustrates how the zero temperature experimental data in (**b**) was determined by Kondo *et al.*^5^. The blue and green areas represent pseudogap and AN spectral weight, respectively. Blue and green double-headed arrows to the left of the vertical axis show the zero temperature magnitude of the pseudogap and AN spectral weight differences, respectively. Black arrows indicate the onset of temperature scales *T*_*AN*_ and T^*^.

Gapped spectral weight is useful when comparing systems at different critical temperatures (or pressure, magnetic field or other parameters) involving phase transitions between states. In this connection, we compare our model to a recent angle-resolved photoemission spectroscopy (ARPES) study of underdoped Bi2201^5^, where the relative strength of two different transitions (pseudogap and antinodal pairing) was determined as a function of doping and temperature^5^. It is interesting to compare their measure of the gapped spectral weight as the depression of spectral weight at 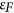) with our spectral weight difference between different ordered states. The Bi2201 ARPES experiment of Ref. 5 found that there was a depression of spectral weight at the Fermi energy, which began to decrease linearly with temperature (*T*) below the pseudogap temperature scale, T^*^(*x*). This was followed by a change in slope to a second value below a scale *T*_*AN*_(*x*), with T^*^ > *T*_*AN*_ > *T*_*C*_, where *T*_*C*_ is the SC critical temperature, as illustrated schematically in Fig. 3(c). *T*_*AN*_ signals the onset of SC pair fluctuations in the antinodal region near (*π*,0), determined at the point on the FS nearest to the antinode. The depression of spectral weight which follows the linear trend down from T^*^ is defined as the contribution to the gapped spectral weight due to the pseudogap, or the *pseudogap spectral weight*. The depression of spectral weight which, beginning at *T*_*AN*_, deviates from the linear trend is defined as the *AN pair spectral weight*. In order to compare these results with our model, we analyze the *T* = 0 limits of our measure of gapped spectral weight at the momentum point on the FS of the AF system at (*π*,0) when no electron pocket is present, or closest to (*π*,0) when the pocket emerges. The calculated pseudogap spectral weight in our model is estimated as the change in spectral weight between the PM and AF phase (defined as the AF state at *T* = 0 with SC order suppressed). The AN pair spectral weight is defined as the difference in spectral weight, at the momentum points stated above, between the AF state and the zero temperature system with AF + SC order.

Our model predicts that at low doping the dominant gapped spectral weight is predominantly associated with the pseudogap spectral weight (blue curve in Fig. 3(a)), but this weight drops suddenly as AN pair spectral weight (green curve in Fig. 3(a)) turns on near TT1 (*x*_*TT*1_ ≃ 0.13), in good agreement with the aforementioned estimates of TT1. Referring to the doping scale at the top of Fig. 3, TT1 has been found in Bi2201 from thermopower^8^ near *x* = 0.166 (green dashed line in Fig. 3), and in STM^22^ (orange dot-dashed line) near *x* = 0.19. The experimental data^5^ display similar steps in both pseudogap (blue symbols in Fig. 3(b)) and AN pair spectral weight (green symbols in Fig. 3(b)) near the same doping, arrow in Fig. 3(b). However, the size of the step is much smaller, and the AN pair spectral weight remains small in most of the underdoped (UD) regime, then increases sharply to a peak slightly above optimal-doping (OPT), decreasing finally in the overdoped (OD) regime. This second transition seen in ARPES^5^ may be associated with a different *topological transition*, possibly related to a competing charge density wave (CDW) order not captured by the present model^12^,^13^,^14^,^23^,^24^,^25^,^26^,^27^,^28^.

**Figure 4:**
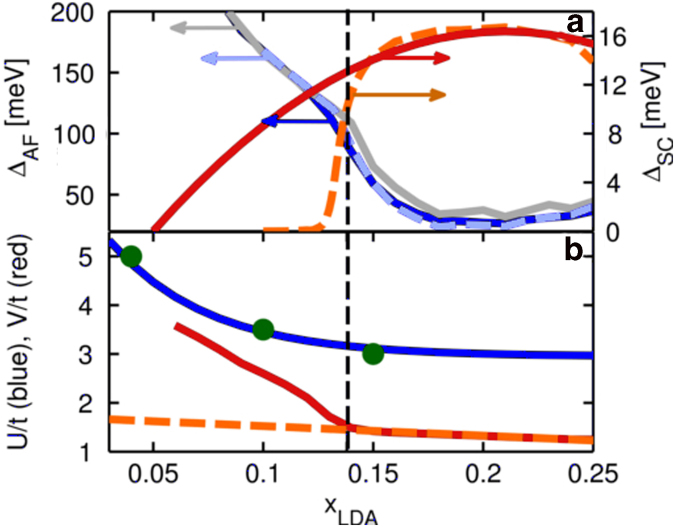
Doping dependence of order parameters and the corresponding potentials. (**a**) Self-consistent values of Δ_*AF*_ as a function of doping for a system with AF order only (gray) or with combined SC + AF order (blue). The red curve shows the SC gap with the scale on the right hand vertical axis. The black dashed line indicates TT1 for our model at *x*_*LDA*_ = 0.138. (b) *U*/*t* fit (blue curve) to the results from Ref. 3 (green circles) as a function of doping and *V*/*t* (red curve) calculated with equation (5) from the assumed SC dome. For the present analysis we are only interested in dopings greater than *x* = 0.05, where the fit is quite good. The orange and light blue dashed curves in (**a**) and (**b**) represent the same quantities as their red and blue, solid lined counterparts, respectively, except that the doping dependence of *V* is assumed linear and Δ_*SC*_ and *S* are calculated using Eqs. 5 and 6. This shows that a large potential *V* is needed for SC order to be sustained to dopings well below the TT1.

It is interesting to examine how TT1 modifies other properties of the cuprates, leading to possible experimental signatures. Figure 4(a) shows that the self-consistent Δ_*AF*_ drops sharply across the transition (vertical dashed line) as the electron pocket opens up. Note that in order to reproduce the experimental SC dome in the low-doping regime, the interaction parameter *V* in Fig. 4(b) must increase rapidly with underdoping below TT1. While a strong increase of the pairing potential near half-filling has been predicted^29^, the dashed line in Fig. 4(b) indicates the effects of a more modest increase in *V*. *T*_*C*_ now decreases very rapidly below the TT1, Fig. 4(a), but there is still a range of FS-free SC in Fig. 2(c). To explain the lower part of the experimental SC dome in this scenario, we would have to postulate that the uniform AF + SC phase becomes unstable to nanoscale phase separation (NPS)^30^,^31^,^32^, which is sensitive to impurities. It could thus lead to the observed low-energy spin-glass phase and to the opening of an additional nodal gap^31^. Termination of this NPS at TT1 suggests that at this doping SC order stabilizes the associated (*π*,*π*) AF order.

Our results fit reasonably within the putative complex picture of pseudogap phenomena in the cuprates. Our analysis indicates that YBCO would harbor four distinct doping regimes within the SC dome. The commensurate (*π*,*π*) AF order at very low doping crosses over to a regime of spin-density wave (SDW)/stripe order, then to a regime of CDW order, and finally to a Fermi-liquid regime. Within each regime there may be further T-dependent phase-mixing, with nearly pure phases occurring only at a few special dopings. Our model is designed for the SDW regime, and we suspect that the sudden onset of SC order at TT1 stabilizes this doping and drives the NPS at lower dopings^31^.

## Discussion

We have shown that FS-free SC, previously observed in the pnictides^1^,^2^, can also occur in hole-doped cuprates. This occurs near the doping at which the topology of the FS changes as an electron pocket appears in the antinodal region, similar to the case of electron doped cuprates^3^,^4^. The resulting spectral weight loss in the SC state is similar to that found in the related ARPES measurements^5^. Our results provide evidence for the presence of two topological transitions under the SC dome in Bi2201, consistent with the picture of a trisected dome in Bi2212^33^.

## Methods

For a given doping *x*, we determine 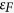 and *S* self-consistently by using Luttinger's theorem to obtain 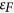 from *x*, and *S* from Eq. (6). The results are shown in Fig. 4. The Hubbard *U*(*x)* is taken as a screened Coulomb potential, which has been studied extensively^3^,^34^,^35^, Fig. 4(b). For this study we used the data for the effective *U*/*t* calculated in Ref. 3which was fit to a decaying exponential 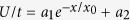, where *a*_1_ = 4.6263, *a*_2_ = 2.95, and *x*_0_ = 0.045. For SC order, we assume that Δ_0_ forms a parabolic dome in doping^36^ with maximum at *x* = 0.21 based on fits of the Fermi energy to experiment^28^. The SC dome is taken to terminate at the VHS^37^. This gives a SC dome which starts at *x* = 0.05, peaks at *x* = 0.21, and terminates at *x* = 0.37. Eq. (5) then determines *V*. However, experimental data are often described in terms of a ‘universal superconducting dome’ (USD) with optimal *T*_*C*_ at *x*_*USD*_ = 0.16 38. This is the case for the Bi2201 ARPES experiment^5^ with which we compare our analysis in Fig. 3. For this comparison we define two doping scales *x*_*LDA*_ and *x*_*USD*_. *x*_*LDA*_ describes the doping determined from our model and *x*_*USD*_ describes doping obtained from the experimental data described in terms of the USD. The transformation of *x*_*USD*_ to *x*_*LDA*_ is given by *x*_*LDA*_ = (32/22)*x*_*USD*_ −0.022727. *x*_*LDA*_ is named as such because tight-binding parameters are often fit to Local Density Approximation (LDA) calculations, although here we take these from fits to experimental data^21^. Finally, we note that the mean-field model provides a good approximation for the low-energy (coherent) dressed states obtained within our intermediate-coupling (QP-GW^16^) model for treating correlation effects in the cuprates. Accordingly, we have matched the experimental dispersion to that of dressed LDA bands at low energies via a dispersion renormalization factor *Z*. The renormalized bands result in the relationship 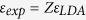 where 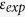 are the bands fit to experiment and 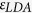 are bands fit to LDA calculations. When considering magnetic order *Z* renormalizes the magnetic susceptibility *X*_0_ and the Hubbard *U*. This leads to 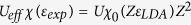, where 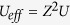 is the effective *U* and 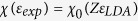 is the renormalized susceptibility. The resulting Stoner criterion is 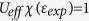.

## Additional Information

**How to cite this article**: Mistark, P. *et al.* Fermi-surface-free superconductivity in underdoped (Bi,Pb)(Sr,La)_2_CuO_6+δ_ (Bi2201). *Sci. Rep.* **5**, 09739; doi: 10.1038/srep09739 (2015).
